# Supplementary material for: S'Wipe: user-friendly stool collection for high-throughput gut metabolomics and multi-omics
Source: mSystems. 2026 Mar 12;11(4):e01459-25. doi: 10.1128/msystems.01459-25 (PMC13098201; doi:10.1128/msystems.01459-25)
Supplement: Legends — for supplemental tables. [file msystems.01459-25-s0002.docx]

**Supplemental Tables and Excel sheets.**

Supplemental Table 1. **Comparison of three collection methods**. Coefficient of variation (CV is listed for each method for three SCFAs of primary diagnostic interest). The average mass of collected specimen (Total (ug/g)) is given.

Supplemental Table 2. The P value SD within stool samples, and SD S'Wipe for each SCFA.

Supplemental Table 3**.** Standard deviation for Neg 80, Across US and intrastate handling conditions.

Supplemental Table 4. Principal coordinate analysis results for comparisons of S’Wipe, direct collection and OMNIgene Gut.

Supplemental Table 5. Coefficient of Variation, Standard deviation LOD/LOQ (µg/mL) and Curve based LOD/LOQ (µg/mL) results for stability of ten SCFAs at room temperature, 40 °C, 4 °C, and −20 °C over 0, 1, 2, 3, 12, 21, and 30 days.

Supplemental Table 6. Comparison of extraction reproducibility across different ethanol concentrations. The coefficient of variation is shown for metabolites extracted with 55%, 60%, and 65% ethanol.

Supplemental Table 7. Coefficient of Variation results of seven SCFAs for 129 samples to reveal reproducible performance across diverse populations.

Supplemental Table 8. P-values for acetic acid, butanoic acid and propanoic acid comparing different collection methods. Scooping and S’Wipe methods are more similar and OMNIgene is clearly different from the other two methods.

Supplemental Excel sheet. **Data Microscopy**. The CellsBin assay for Samples 1,2, 3, 4, 5,6, 10, 11, and 12.
