# Supplementary material for: S'Wipe: user-friendly stool collection for high-throughput gut metabolomics and multi-omics
Source: mSystems. 2026 Mar 12;11(4):e01459-25. doi: 10.1128/msystems.01459-25 (PMC13098201; doi:10.1128/msystems.01459-25)
Supplement: Table S1 — Comparison of three collection methods. [file msystems.01459-25-s0003.docx]

|  | OMNIgene™ | Direct collection | S'Wipe™ |
| --- | --- | --- | --- |
| Acetic acid | **8.9%** | **13.4%** | **11.8%** |
| Butanoic acid | **26.3%** | **22.9%** | **17.8%** |
| Propanoic acid | **17.6%** | **15.6%** | **12.5%** |
| Total (ug/g) | **267.6** | **112.4** | **355.0** |
