# Supplementary material for: S'Wipe: user-friendly stool collection for high-throughput gut metabolomics and multi-omics
Source: mSystems. 2026 Mar 12;11(4):e01459-25. doi: 10.1128/msystems.01459-25 (PMC13098201; doi:10.1128/msystems.01459-25)
Supplement: Table S2 — The P value SD within stool samples and SD S'Wipe for each SCFA. [file msystems.01459-25-s0004.docx]

| Metabolite | p-value | SD-Within Stool samples (AUC a.u.) | Mean Values Stool samples (AUC a.u.) | SD-Within SWipe samples (AUC a.u.) | Mean Values SWipe samples (AUC a.u.) |
| --- | --- | --- | --- | --- | --- |
| Acetic acid | 0.57 | 1511.973186 | 3873.199 | 2079.45994 | 4467.433 |
| Propanoic acid | 0.5 | 248.1220379 | 827.881 | 466.316438 | 973.963 |
| Isobutyric acid | 0.64 | 103.488392 | 352.296 | 182.4570342 | 392.660 |
| Butanoic acid | 0.52 | 460.6492991 | 1356.358 | 676.8310287 | 1568.428 |
| Isovaleric acid | 0.80 | 268.704114 | 645.128 | 274.7302458 | 682.099 |
| Pentatonic acid | 0.8 | 423.7636601 | 784.026 | 279.7581229 | 731.178 |
| Isocaproic acid | 0.4 | 544.4020334 | 522.337 | 216.7319535 | 337.243 |
| Hexanoic acid | 0.97 | 618.8879559 | 1155.621 | 393.4358871 | 1163.049 |
| Heptanoic acid | 0.4 | 957.1050945 | 1032.117 | 332.0338515 | 699.002 |
| p-Cresol | 0.74 | 132.1126901 | 356.179 | 133.8460028 | 332.3452 |
| Total SCFA | 0.8 | 4136.47976 | 10905.14 | 4473.750664 | 11347.402 |
