# Supplementary material for: S'Wipe: user-friendly stool collection for high-throughput gut metabolomics and multi-omics
Source: mSystems. 2026 Mar 12;11(4):e01459-25. doi: 10.1128/msystems.01459-25 (PMC13098201; doi:10.1128/msystems.01459-25)
Supplement: Table S3 — Standard deviation for Neg 80, Across US, and intrastate handling conditions. [file msystems.01459-25-s0005.docx]

| Metabolite | Mean Values for Intrastate | SD for Intrastate | Mean Values for Across US | SD for Across US | Mean Values for Neg 80 | SD for Neg 80 |
| --- | --- | --- | --- | --- | --- | --- |
| Acetic acid | 5.2 | 1.12 | 7.0 | 0.525 | 4.1 | 0.213 |
| Propanoic acid | 2.0 | 0.406 | 2.2 | 0.212 | 1.7 | 0.190 |
| Isobutyric acid | 0.70 | 0.198 | 0.71 | 0.163 | 0.75 | 0.151 |
| Butanoic acid | 1.5 | 0.305 | 1.9 | 0.154 | 1.2 | 0.060 |
| Isovaleric acid | 0.76 | 0.212 | 0.74 | 0.174 | 0.80 | 0.155 |
| Valeric acid | 0.52 | 0.214 | 0.57 | 0.075 | 0.54 | 0.053 |
| Isocaproic acid | 0.66 | 0.241 | 0.63 | 0.193 | 0.73 | 0.184 |
| Caproic acid | 1.14 | 0.414 | 1.15 | 0.258 | 1.25 | 0.322 |
| Heptanoic acid | 0.82 | 0.300 | 0.77 | 0.242 | 0.91 | 0.230 |
| Phenol | 0.49 | 0.185 | 0.45 | 0.140 | 0.53 | 0.136 |
| p-Cresol | 0.41 | 0.156 | 0.37 | 0.115 | 0.42 | 0.099 |
| Total SCFA | 13.4 | 2.89 | 15.59 | 1.342 | 12.05 | 1.29 |
