# Supplementary material for: S'Wipe: user-friendly stool collection for high-throughput gut metabolomics and multi-omics
Source: mSystems. 2026 Mar 12;11(4):e01459-25. doi: 10.1128/msystems.01459-25 (PMC13098201; doi:10.1128/msystems.01459-25)
Supplement: Table S4 — Principal coordinate analysis results for comparisons of S'Wipe, direct collection, and OMNIgene Gut. [file msystems.01459-25-s0006.docx]

|  | **Adonis2 Pairwise comparison** | **group size** | **F.Model** | **R2** | **nperm** | **FDR** |
| --- | --- | --- | --- | --- | --- | --- |
| 1 | S'wipe vs Regular | 9−9 | 1.05 | 0.06 | 999 | 0.319 |
| 2 | S'wipe vs OmniMET | 9−9 | 1.78 | 0.10 | 999 | 0.003 |
| 3 | Regular vs OmniMET | 9−9 | 1.56 | 0.09 | 999 | 0.003 |
