# Supplementary material for: S'Wipe: user-friendly stool collection for high-throughput gut metabolomics and multi-omics
Source: mSystems. 2026 Mar 12;11(4):e01459-25. doi: 10.1128/msystems.01459-25 (PMC13098201; doi:10.1128/msystems.01459-25)
Supplement: Table S5 — Coefficient of variation, standard deviation LOD/LOQ, and curve-based LOD/LOQ results for stability of ten SCFAs at different temperatures and times. [file msystems.01459-25-s0007.docx]

| Metabolite | CV | Standard deviation LOD/LOQ (µg/mL) | Curve based LOD/LOQ (µg/mL) |
| --- | --- | --- | --- |
| Acetic acid | 0.172 | 0.20 / 0.68 | 0.015 / 0.049 |
| Propanoic acid | 0.173 | 0.17 / 0.56 | 0.019 / 0.063 |
| Isobutyric acid | 0.198 | 0.15 / 0.48 | 0.052 / 0.173 |
| Butanoic acid | 0.163 | 0.21 / 0.71 | 0.017 / 0.058 |
| Isovaleric acid | 0.158 | 0.15 / 0.49 | 0.015 / 0.050 |
| Valeric acid | 0.144 | 0.24 / 0.79 | 0.019 / 0.063 |
| Phenol | 0.201 | 0.01 / 0.05 | 0.055 / 0.184 |
| p-Cresol | 0.156 | 0.02 / 0.05 | 0.055 / 0.184 |
| Indole | 0.161 | 0.01 / 0.03 | 0.040 / 0.135 |
| Skatole | 0.137 | 0.01 / 0.04 | 0.063 / 0.209 |
