# Supplementary material for: S'Wipe: user-friendly stool collection for high-throughput gut metabolomics and multi-omics
Source: mSystems. 2026 Mar 12;11(4):e01459-25. doi: 10.1128/msystems.01459-25 (PMC13098201; doi:10.1128/msystems.01459-25)
Supplement: Table S6 — Comparison of extraction reproducibility across different ethanol concentrations. [file msystems.01459-25-s0008.docx]

| Metabolite | CV_55% | CV_60% | CV_65% | ANOVA_p |
| --- | --- | --- | --- | --- |
| Acetic acid | 4.96 | 6.88 | 8.10 | 0.01 |
| Propanoic acid | 5.95 | 6.44 | 8.48 | 0.3 |
| Isobutyric acid | 8.34 | 7.03 | 9.26 | 0.03 |
| Butanoic acid | 6.10 | 6.50 | 8.61 | 0.6 |
| Isovaleric acid | 10.22 | 22.06 | 19.84 | 0.2 |
| Valeric acid | 8.32 | 7.02 | 9.07 | 0.05 |
| Phenol | 6.80 | 7.64 | 8.42 | 0.01 |
| p-Cresol | 6.90 | 7.35 | 8.98 | 0.11 |
| Indole | 6.10 | 6.65 | 10.18 | 0.001 |
| Skatole | 5.99 | 7.058 | 9.24 | 0.001 |
