# Supplementary material for: S'Wipe: user-friendly stool collection for high-throughput gut metabolomics and multi-omics
Source: mSystems. 2026 Mar 12;11(4):e01459-25. doi: 10.1128/msystems.01459-25 (PMC13098201; doi:10.1128/msystems.01459-25)
Supplement: Table S7 — Coefficient of variation results of seven SCFAs for 129 samples to reveal reproducible performance across diverse populations. [file msystems.01459-25-s0009.docx]

| Metabolite | CV |
| --- | --- |
| Acetic acid | 1.341 |
| Butanoic acid | 1.736 |
| Propanoic acid | 1.96 |
| p-Cresol | 2.062 |
| Indole | 1.488 |
| Skatole | 2.154 |
| Phenol | 1.008 |
