# Supplementary material for: S'Wipe: user-friendly stool collection for high-throughput gut metabolomics and multi-omics
Source: mSystems. 2026 Mar 12;11(4):e01459-25. doi: 10.1128/msystems.01459-25 (PMC13098201; doi:10.1128/msystems.01459-25)
Supplement: Table S8 — P values for acetic acid, butanoic acid, and propanoic acid comparing different collection methods. [file msystems.01459-25-s0010.docx]

| Comparison | P-value for Acetic acid | P-value for Butanoic acid | P-value for Propanoic acid |
| --- | --- | --- | --- |
| OMNIgene vs. Scooping | 0.00229 | 0.00304 | 0.00375 |
| OMNIgene vs. S’Wipe | 0.00008 | 0.00007 | 0.00541 |
| Scooping vs. S’Wipe | 0.30425 | 0.19096 | 0.58899 |
